# An Account of the Examination of the Body of a Little Boy, Who Died of the Hydrophobia; Intended to Show the Probable Success of Dr. Physick's Proposal for Preventing Death by Making an Artificial Opening into the Windpipe

**Published:** 1808-10

**Authors:** Benjamin Rush

**Affiliations:** Professor of Medicine in the University of Pennsylvania


### *An Account of the Examination of the Body of a little Boy, who died of the* HYDROPHOBIA; *intended to show the probable Success of Dr.* PHYSICK'S *Proposal for preventing Death by making an artificial Opening into the Windpipe. By* BENJAMIN RUSH, *M. D. Professor of Medicine in the University of Pennsylvania.*

IN the first number of the fifth volume of the American Medical Repository, Dr. Physick has supposed death from Hydrophobia to be the effect of a sudden and spasmodic constriction of the glottis, inducing suffocation, and that it might be prevented by creating an artificial passage for air air into the lungs, whereby life might be continued long enough to admit of the disease being cured by other remedies. The following account of a dissection is intended to show the probability of the Doctor's proposal being attended with success.

On the 13th of September, 1802, I was called, with Dr. Physick, to visit, in consultation with Dr. Griffitts, the son of William Todd, Esq. aged five years, who was ill with the disease called Hydrophobia, brought on by the bite of a mad dog, on the 6th of the preceding month. The wound was small, and on his cheek, near his mouth; two circumstances which are said at all times to increase the danger of wounds from rabid animals. From the time he was bitten he used the cold bath daily, and took the infusion, powder, and seeds of the Anagallis, in succession, until the 9th of September, when he was seized with a fever, which at first resembled the remittent of the season. Bleeding, purging, blisters, and the warm bath were prescribed for him, but without success. The last named remedy appeared to afford him some relief, which he manifested by paddling and playing in the water. At the time I saw him he was much agitated, had frequent twitchings, laughed often; but with this uncommon excitement in his muscles and nerves, his mind was unusually correct in all its operations.

He discovered no dread of water except in one instance, when he turned from it with horror. He swallowed occasionally about a spoonful of it at a time, holding the cup in his own hand, as if to prevent too great a quantity being poured at once into his throat. The quick manner of his swallowing, and the intervals between each time of doing so, were such as we sometimes observe in persons in the act of dying of acute diseases. Immediately after swallowing water, he looked pale, and panted for breath. He spoke rapidly, and with much difficulty. This was more remarkably the case when he attempted to pronounce the words Carriage, Water, and River. After speaking he panted for breath in the same manner that he did after drinking. He coughed and breathed as patients do in the moderate grade of the cynanche trachealis. The dog that had bitten him, Mr. Todd informed me, made a similar noise in attempting to bark a day or two before he was killed. We proposed making an opening into his windpipe. To this his parents readily consented; but while we were preparing for the operation, such a change for the worse took place, that we concluded not to perform it. A

cold sweat, with a feeble and quick pulse, came on; and he died suddenly, at 12 o'clock at night, about six hours after I first saw him. He retained his reason, and a playful humour, till the last minute of his life. An instance of the latter appeared in his throwing his handkerchief at his father just before he expired.

The parents consented to our united request to examine his body. Dr. Griffitts being obliged to go into the country, and Dr. Physick being indisposed, I undertook this business the next morning; and, in the presence of Dr. John Dorsey, (to whom I gave the dissecting knife) and Mr. Murduck, I discovered the following appearances:— All the muscles of the neck had a livid colour, such as we sometimes observe, after death, in persons who have died of the sore throat. The muscles employed in deglutition and speech were suffused with blood. The epiglottis was inflamed, and the glottis so thickened and contracted as barely to admit a probe of the common size. The trachea below it was likewise inflamed and thickened, and contained a quantity of mucus in it, such as we observe now and then, after death, from cynanche trachealis. The œsophagus exhibited no marks of disease; but the stomach had several inflamed spots upon it, and contained a matter of a brown appearance, and which emitted an offensive odour.

From the history of this dissection, and of many others in which much fewer marks appeared of violent disease in parts whose actions are essential to life, it is highly probable that death is not induced in the ordinary manner in which malignant fevers produce it, but by a sudden or gradual suffocation. It is the temporary closure of this aperture which produces the dread of swallowing liquids: hence the reason why they are swallowed suddenly, and with intervals, in the manner that has been described; for, should the glottis be closed during the time of two swallows, in the highly inflamed state of the system which takes place in this disease, suffocation would be the immediate and certain consequence. The same difficulty and danger attend the swallowing saliva, and hence the symptom of spitting, which has been so often taken notice of in Hydrophobia. Solids are swallowed more easily than fluids, only because they descend by intervals, and because a less closure of the glottis is sufficient to favour their passage into the stomach. This remark is confirmed by the frequent occurrence of death in the very act of swallowing, and that too with the common symptoms of suffocation.

tion. To account for death from this cause, and in the manner that has been described, it will be necessary to recollect that fresh air is more necessary to the action of the lungs in a fever than in health, and much more so in a fever of a malignant character, such as the Hydrophobia appears to be, than in fevers of a milder nature. An aversion from swallowing liquids is not peculiar to this disease. It occurs occasionally in the yellow fever. It occurs likewise in the disease which has prevailed among the cats both in Europe and America, and probably in both instances from a dread of suffocation in consequence of the closure of the glottis, and sudden abstraction of fresh air.

The seat of the disease, and the cause of death, being, I hope, thus ascertained, the means of preventing death come next under our consideration. Tonic remedies, in all their forms, have been administered to no purpose. The theory of the disease would lead us to expect a remedy for it in blood-letting. But this, though now and then used with success, is not its cure; owing, as we now see, to the mortal seat of disease being so far removed from the circulation as not to be affected by the loss of blood in the most liberal quantity. As well might we expect the inflammation and pain of a paronychia, or what is called a felon on the finger, to be removed by the same remedy. Purging and sweating, though occasionally successful, have failed in many instances; and even a salivation, when excited, (which is rarely the case) has not cured it. An artificial aperture into the windpipe alone bids fair to arrest its tendency to death, by removing the symptom which generally induces it, and thereby giving time for other remedies, which have hitherto been unsuccessful, to produce their usual salutary effects in similar diseases.* In removing faintness, in drawing off the water in ischuria, in composing convulsions, and in stopping hæmorrhages in malignant fever, we do not cure the disease, but we prevent death, and thereby gain time for the use of the remedies which are proper to cure it. Laryngotomy, according to Fourcroy's advice, in diseases of the throat which obstruct respiration, should be preferred to tracheotomy, and the incision should be made in the triangular space between the thyroid and cricoid cartilages. Should this operation be

* The hoarse barking, or the total inability of mad dogs to bark, favours still further the idea that the mortal seat of the disease is in the glottis, and that the remedy which has been proposed is a rational one.

be adopted in order to save life, it will not offer near so much violence to humanity as many other operations. We cut through a large mass of flesh into the bladder in extracting a stone. We cut into the cavity of the thorax in the operation for the empyema. We perforate the bones of the head in trepanning; and we cut through the uterus, in performing the Cæsarian operation, in order to save life. The operation of laryngotomy is much less painful and dangerous than any of them; and besides permitting the patient to breathe and to swallow, it is calculated to serve the inferior purpose of lessening the disease of the glottis by means of local depletion. After an aperture has been thus made through the larynx, the remedies should be such as are indicated by the state of the system, particularly by the state of the pulse. In hot climates it is, I believe, generally a disease of feeble re-action, and requires tonic remedies; but in the middle and northern States of America it is more commonly attended with so much activity and excitement of the blood vessels, as to require copious blood-letting and other depleting remedies.

Should this new mode of attacking this furious disease be adopted, and become generally successful, the dicovery will place the ingenious gentleman who suggested it in the first rank of the medical benefactors of mankind.

Before I conclude my letter, I have only to add a fact which may tend to increase confidence in a mode of preventing the disease, which has been recommended by Dr. Haygarth, and used with success in several instances. The same dog which bit Mr. Todd's son, bit, at the same time, a cow, a pig, a dog, and a black servant of Mr. Todd's. The cow and pig died; the dog became mad, and was killed by his master. The black man, who was bitten on one of his fingers, exposed the wound for some time, immediately after he received it, to a stream of pump-water, and washed it likewise with soap and water. He happily escaped the disease, and is now in good health. That his wound was poisoned is highly probable, from its having been made eight hours after the last of the above animals was bitten, in which time there can be but little doubt of such a fresh secretion of saliva having taken place as would have produced the hydrophobia, had it not been prevented by the above simple remedy. I am not, however, so much encouraged by its happy issue in this case as to advise it in preference to cutting out the wounded part. It should only be resorted to where the fears of a patient, or his distance from a surgeon, render it impossible to use the knife.

ROYAL

